# Polyphenols from *Ginkgo biloba*

**DOI:** 10.3797/scipharm.1003-19

**Published:** 2010-10-28

**Authors:** Fadi Qa’dan, Adolf Nahrstedt, Mathias Schmidt, Kenza Mansoor

**Affiliations:** 1 Faculty of Pharmacy, Petra University, Airport Street, P O Box 961343 Amman, Jordan; 2 Institut fuer pharmazeutische Biologie und Phytochemie, Hittorfstrasse 56, D-48149 Muenster, Germany; 3 HerbResearch, Wartbergweg 15, D-86874 Mattsies, Germany; 4 School of Pharmacy, Al-Ahliyya Amman University: Amman-19328, Jordan

**Keywords:** *Ginkgo biloba*, Polymeric proanthocyanidins, Anti oxidant, Fremy’s salt

## Abstract

By Sephadex LH-20 gel chromatography of an extract from *Gingko biloba* leaves, polymeric proanthocyanidins were eluted after the fractions of flavonol glycosides and biflavone glycosides. A purified proanthocyanidin polymer accounted for 86.6% of the total proanthocyanidins, and for 37.7% of the total antioxidant activity of this leaf extract. For structure elucidation, the polymer was submitted to acidic depolymerization in the presence of phloroglucinol. The structures of the resulting flavan-3-ols and phloroglucinol adducts were determined on the basis of 1D-and reverse 2D-NMR (HSQC, HMBC) spectra of their peracetylated derivatives, MALDI-TOF-MS and CD-spectroscopy. The observations resulting from the degradation with phloroglucinol were confirmed by ^13^C-NMR spectroscopy of the polymer. The mean molecular weight of the polymeric fraction was estimated to be 9–10 flavan-3-ol-units.

## Introduction

Extracts of *Ginkgo biloba* L. (Maidenhair tree, Ginkgoaceae) are widely used in herbal medicine for the treatment of mild to moderate cognitive disorders, concentration problems, tinnitus and dementia [1]. Ginkgo extracts are also used against Alzheimer’ disease [2]. Most of the extracts are standardized to a typical composition of 6% terpene trilactones, 24% of flavonol glycosides and less than 5 ppm of ginkgolic acids [3, 4]. Ginkgo leaves and standardized extracts contain large amounts of proanthocyanidins (PAC, 4–12% and 7% in leaves and extracts, respectively) [5, 6]. In spite of their quantitative importance, relatively little is known about the composition of the PAC in Ginkgo. The only compounds identified to date are dimeric procyanidins and prodelphinidins [7]. The knowledge of the phytochemical composition of the polymeric proanthocyanidin fraction (acetone-water-eluted fraction, AWF) is, however, of importance for the understanding of the relation between the chemical structure and the antioxidant activity of Ginkgo extracts.

Our study focused on the isolation and characterization of a fraction containing polymeric proanthocyanidins (AWF) from *Ginkgo biloba* by degradation with phloroglucinol and identification of the break-down products

## Results and Discussion

A defatted crude acetone/water (7:3) extract of *Ginkgo biloba* leaves was repeatedly extracted with ethyl acetate to remove the terpenoids, flavonoids, isoflavonoids, phenolic acids, alkyl phenols and the lower oligomeric proanthocyandins. The remaining water-soluble residue was further fractionated on an LH-20 Sephadex column with methanol-water (1:1) as a mobile phase to separate carbohydrates, relatively polar flavonoids and the higher oligomeric proanthocyanidns from polymeric condensed tannins [8]. The AWF fraction was then eluted with acetone-water (7:3).

In order to distinguish the compounds relevant for the antioxidant activity of *Ginkgo biloba* leaf extract, the ethyl acetate fraction (EAF), the methanol-water fraction (MWF) and the acetone-water eluted fraction (AWF) were investigated by ESR spectroscopy (Tab. 1). The antioxidant activity of the three fractions (EAF, MWF, AWF) was expressed as its ability to reduce a synthetic free radical species obtained from the reaction with Fremy’s salt [9].

By HPLC and TLC-examinations we could demonstrate that the EAF contains mainly terpenoids, flavonoids, flavan-3-ols, lower oligomeric proanthocyanidins and other phenolic compounds. The MWF contains mainly carbohydrates, flavonol glycosides and higher oligomeric proanthocyanidins.

The EAF and the MWF possess relatively strong antioxidant properties (Tab. 1.), most likely related to the excellent radical scavenging properties of the ginkgolides, biflavones and the flavonol glycosides. 37.7 % of the total antioxidant activity of *Ginkgo biloba* crude extract was detected in the AWF – the fraction which also contained the highest proanthocyanidin concentration. Thus, our objective was to obtain detailed structural information on this proanthocyanidin fraction. The AWF fraction (for details see experimental part) showed an optical rotation of +81° (c 0.1, MeOH), which corresponds to a molar proportion of subunits with a relative 2,3-*cis* stereochemistry of 83.5 % [10]. By integration of the ^13^C NMR-signals close to δ = 77 ppm and δ = 84 ppm (solvent: MeOH-d_4_, 99 MHz), an approximately 4.5:1 ratio was obtained for *cis : trans* isomers [11, 12]. The ratio of the signal intensities at (δ = 115-116 ppm) and at 107 ppm revealed that the AWF fraction is composed of approximately 15 % of procyanidin (PC) and 85 % of prodelphinidin (PD) [12]. The mean average molecular size of the polymeric proanthocyanidin in the AWF fraction was estimated to be 9–10 flavan-3-ol units by integration of the C-3 signals of the extender units at 73 ppm and the corresponding signal of the lower flavan-3-ols at 68 ppm [13].

In order to elucidate the structure in more detail, the AWF fraction was degraded in the presence of phloroglucinol under acidic conditions at ambient temperature for 30 min [13, 14]. The reaction resulted in the cleavage of the terminal flavanoid units, which were identified as epigallocatechin in relatively high amounts, catechin, gallocatechin, and relatively low quantities of epicatechin. Among the monomeric phloroglucinol-captured products, epigallocatechin-(4β→2)-phloroglucinol was isolated as the main monomeric adduct in addition to small amounts of epicatechin-(4β→2)-phloroglucinol and gallocatechin-(4α→2)-phloroglucinol. The overall ratio of monomers is consistent with the ^13^C NMR data. The structures of the flavan-3-ols and the monomeric phloroglucinol adducts were identified on the basis of 1D- and 2D-NMR (HSQC, HMBC) experiments of their peracetylated derivatives. Comparison of the data with authentic samples from earlier work and with published values allowed the identification of these compounds [16–23].

Some larger scission products containing identifiable interflavonoid linkages had to be isolated for the establishment of the nature of the such bonds in the AWF fraction. Five compounds were isolated by column chromatography alternating between Sephadex LH-20 and high porosity polystyrene polymer (MCI CHP 20P), using aqueous methanol as a mobile phase. The dimeric phloroglucinol adduct epigallocatechin-(4β→8)-epigallocatechin-(4β→2)-phloroglucinol (compound **1**) was isolated as the main dimer in addition to epigallocatechin-(4β→6)-epigallocatechin-(4β→2)-phloroglucinol (compound **2**), epigallocatechin-(4β→8)-catechin, epigallocatechin-(4β→8)-epigallocatechin and small amounts of the prodelphinidin epigallocatechin-(4β→8)-gallocatechin.

**Fig. 1.**
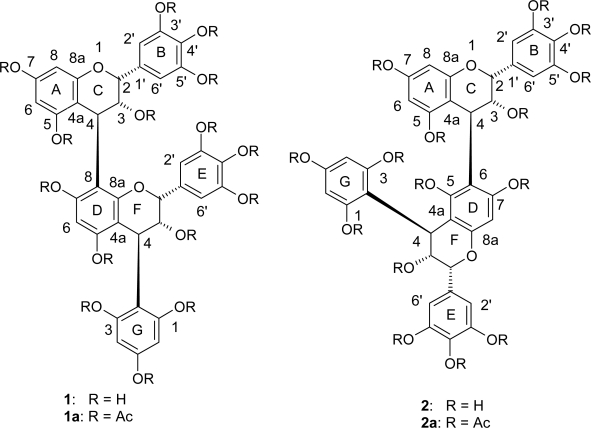
Structural features of the dimeric phloroglucinol adducts **1** and **2**

The structure of compounds **1** and **2** (Fig. 1.) were determined on the basis of their 1D-and 2D-NMR (HSQC, HMBC), CD and [α]^20^ data of their peracetylated derivatives. The structures of epigallocatechin-(4ß→8)-catechin, epigallocatechin(4β→8)-epigallocatechin and epigallocatechin-(4β→8)-gallocatechin were identified by ^1^H-NMR, MALDI-TOF-MS and CD spectroscopy of the peracetylated derivative in comparison with published data [19–24].

Compound **1** showed a prominent quasi-molecular ion peak at *m*/*z* 1384 [M+Na]^+^ in the MALDI-TOF-MS of its peracetate (compound **1a**), which suggests a B-type diflavanoid constitution composed of two (epi)gallocatechin units and one additional phloroglucinol ring. The ^1^H-NMR spectrum at ambient temperature showed complex signal duplication due to rotational isomerism. The assignment of signals and the point of interflavan-linkage were achieved by extensive 2D-NMR experiments. The spectral pattern of the heterocyclic region of compound **1a** was almost identical with the corresponding procyanidin derivative epicatechin-(4β→8)-epicatechin-(4β→2)-phloroglucinol [17]. The location of the interflavanoid linkages was recognized for **1a** by long-range correlations (HMBC) of H-4 (C) with C-8a (D). This key correlation indicates that the flavan-3-ol units are C-4/C-8 linked [25]. Under the conditions employed, compound **1** gave phloroglucinol and epigallocatechin-(4β→2)-phloroglucinol as the main degradation products in the reaction with 0.1 M ethanolic HCl [27]. These degradation products were identified by co-chromatography in comparison with authentic compounds. The high amplitude positive cotton effect at 200–240 nm in the CD-spectrum of **1a** confirmed the absolute configuration as 4R [25, 26]. In conjunction with the optical rotation [α]^20^ = +99.3° (c 0.10, MeOH), compound **1** was characterized as epigallocatechin-(4β→8)-epigallocatechin-(4β→2)-phloroglucinol.

The structure of epigallocatechin-(4β→6)-epigallocatechin-(4β→2)-phloroglucinol (compound **2**) was established with reverse 2D NMR methods of its peraceatate (compound **2a**). The ^1^H-NMR of **2a** was similar to that of the analogous dimeric prodelphinidin epigallocatechin-(4β→6)-epigallocatechin peracetate [23]. The peracetate of **2a** showed two additional proton doublets of δ 6.76 and 6.88 ppm, respectively, due to the meta-coupling protons of the additional phloroglucinol ring (G-ring). Compound **2** showed a prominent ion peak at *m*/*z* 1384 in the MALDI-TOF-MS [M+Na]^+^ of its peracetate (**2a**), indicative of a dimeric proanthcyanidin derivative composed of two gallocatechin/epigallocatechin and one phloroglucinol ring. The ^1^H-NMR of **2a** in CDCl_3_ (600 MHz) gave two sharp two-proton singlets at δ 7.16 and 7.29 ppm typical for pyrogallol-type-B rings of the constituent flavan-3-ol units. The location of the inter-flavanoid linkage was recognized for **2a** by long-range correlation (HMBC) of H-4 (C) with C-5 (D) [24]. This key correlation indicates that the flavan-3-ol units are C-4/C-6 linked. The heterocyclic coupling constants (*J*_2,3_ < 2 MHz) confirmed the relative 2,3-*cis* configuration of the “upper” and “lower” constituent units [15]. The major degradation products of compound **2** after acid-catalyzed degradation were epigallocatechin-(4β→2)-phloroglucinol and phloroglucinol [27]. These degradation products were identified by co-chromatography in comparison with authentic compounds. The high amplitude positive cotton effect at 200–240 nm in the CD-spectrum of **2a** confirmed the absolute configuration as 4R [25, 26]. In conjunction with the optical rotation [α]^20^ = +179° (c 0.17, MeOH), compound **2** was characterized as epigallocatechin-(4β→6)-epigallocatechin-(4β→2)-phloroglucinol.

To the best of our knowledge, compounds **1** and **2** as well as the NMR-data of their peracetate derivatives are described for the first time.

In conclusion, we found that the AWF fraction accounted for 37.7 % of the total antioxidant activity of *Ginkgo biloba* leaf extract. The flavan-3-ol units in the AWF fraction showed great similarity in the chemical structure to the dimeric procyanidins and prodelphinidins previously identified by paper chromatography and HPLC [7]. In addition, the 2,3-*cis*-configuration and 3’,4’,5’-trihydroxylated B-rings were predominant in the isolated polymer, and 3-*O*-galloylated units were not observed.

Future investigations will deal with the pharmacological testing of the AWF fraction to corroborate their presumed contribution to antioxidant mechanisms and the overall clinical effects of Ginkgo extracts in the treatment of mild to moderate cognitive disorders, tinnitus, and dementia.

## Experimental

### General

^1^H NMR spectra were recorded in CDCl_3_ on a Varian Gemini 200 (200 MHz), on a Varian Mercury 400 plus or a Bruker AM 600 (600 MHz) relative to CHCl_3_. ^13^C-NMR were recorded at 50, 100, and 150 MHz, respectively. CD data were obtained in MeOH on a Jasco J 600. MALDI-TOF mass spectrometer: LAZARUS II (home built), N2-laser (LSI VSL337ND) 337 nm, 3 ns pulse width, focus diameter 0.1 mm, 16 kV acceleration voltage, 1 m drift length, data logging with LeCroy9450A, 2.5 ns sampling time and expected mass accuracy +/− 0.1 %, sample preparation: acetylated compounds were deposited from a solution in CHCl_3_ on a thin layer of 2,5-dihydroxybenzoic acid (DHB) crystals. Analytical TLC was carried out on aluminium sheets (Silica gel 60 F_254_, 0.2 mm, Merck) using acetone-toluene-formic acid (60 : 30 : 10). Compounds were visualized by spraying with vanillin-HCl reagent and 1% ethanolic FeCl_3_ solution. Acetylations were performed in pyridine-acetic acid anhydride (1:1.2) at ambient temperature and a reaction time of 24 h.

### Materials

Phloroglucinol was obtained from Fluka (Seelze, Germany). Potassium nitrosodisulfonate (Fremy’s salt) was purchased from Sigma-Aldrich (Taufkirchen, Germany). Reagents and solvents were purchased from Roth (Karlsruhe, Germany) or Merck (Darmstadt, Germany).

### Plant material

*Ginkgo biloba* L. dried plant material (Ch-B.: 4407655) was obtained from Martin Bauer GmbH, Vestenbergsgreuth, Germany. Identification was performed by microscopic investigations. A voucher specimen is retained in the documentation file of the School of Pharmacy, Petra University, under the code Ginkgo 1.

### Quantitative analysis of proanthocyanidins

The content of proanthocyanidins in the crude extract and the three fractions (EAF, MWF, AWF) was determined photometrically after acidic depolymerization to the corresponding anthocyanidins [9]. In the crude extract and all fractions, 1 mg of the dried sample was dissolved in 10 mL of a solution of concentrated hydrochloric acid in n-butanol (10:90, v/v). The closed vial containing the solution was mixed vigorously and heated for 90 min in a boiling water bath. After the solution was cooled to room temperature, the absorbance at 550 nm was measured using a Novaspec II spectrophotometer (Pharmacia LKB, Uppsala, Sweden). The content of PAC (calculated as mg cyanidin/L) was calculated by the molar extinction coefficient of cyanidin (ɛ = 17 360 L mol^−1^ cm^−1^).

### Electron spin resonance (ESR) analysis

For measuring the antioxidant activity of the crude extract and the three fractions, 1 mg of the crude extract and each fraction was dissolved in 1 mL methanol. Aliquots (500 μL) were allowed to react with an equal volume of Fremy’s salt (1 mM in phosphate buffer pH 7.4). The ESR spectrum of Fremy’s radical was obtained after 20 min, by which time the reaction was completed. Signal intensity was obtained by integration. The antioxidant activity, expressed as molar equivalents of Fremy’s salt reduced by one mol of antioxidant, was calculated by comparison with a control reaction with methanol. Spectra were obtained at 21 °C on a Miniscope MS 100 spectrometer (Magnettech). The microwave power and modulation amplitude were set to 10 dB and 1500 mG, respectively. For the measurement, 50 μL of the reaction mixture was added with a micropipette.

### Extraction and preparation of the polymeric proanthocyanidin fraction

Air-dried material (5 kg) was exhaustively extracted with acetone/water (7: 3, 35 L). The combined extracts were evaporated *in vacuo* to a volume of 2 L, filtered to remove the precipitated chlorophyll, concentrated and defatted with petroleum benzene 30–50 °C (liquid crude extract). A part of the liquid crude extract (10 ml) was evaporated to dryness and freeze-dried to yield 2.2 g dried crude extract. Successive extractions of the remaining liquid crude extract with ethyl acetate (8 L) followed by evaporation of the solvent yielded 33.5 g of solid residue in the ethyl acetate fraction (EAF).

The remaining water-phase was evaporated to dryness (450 g). A portion (4 × 50 g) of the residue from the water extract was successively applied to column chromatography on Sephadex LH-20 (55 × 900 mm) with 27 L MeOH-H_2_O (1:1) until the eluent was colourless (MWF; 168.5 g). Subsequently, acetone/water (3:7; 7 L) was used to elute the polymeric fraction (AWF; 31.5 g).

### Degradation with phloroglucinol and isolation of proanthocyanidin cleavage products

An aliquot of the AWF fraction of *Ginkgo biloba* obtained as described above (18 g) was treated under shaking with phloroglucinol (13.5 g) in 1% HCl in EtOH (100 mL) for 30 min. at room temperature [14]. The solution was concentrated under reduced pressure to give 31.5 g of reaction products (PA degradation fraction).

A portion (25 g) of the PA degradation fraction was fractionated by CC on Sephadex LH-20 (55 × 900 mm) using EtOH (96%) (25 L), EtOH-MeOH 1:1 (6 L) and Me_2_CO-H_2_O 3:7 (3 L) as eluents to give 10 fractions.

Fraction 2 (1430–3610 mL, 170 mg) was subjected to chromatography on MCI-gel CHP 20P (25 × 250 mm) with a 10–80 % MeOH linear gradient (17 mL/fraction) to yield catechin (subfractions 23–46, 51 mg) and epicatechin (subfractions 48–59, 16 mg).

Fraction 3 (3610–4600 mL, 755 mg) was separated on MCI-gel with the same gradient as above to yield gallocatechin (subfractions 15–21, 111 mg) and epigallocatechin (subfractions 25–41, 527 mg).

Fraction 4 (4600–5300 mL, 250 mg) was separated on MCI-gel to yield epicatechin-(4β→2)-phloroglucinol (subfractions 23–31, 77 mg) and catechin-(4α→2)-phloroglucinol (subfractions 52–59, 109 mg).

Fraction 5 (5300–5800 mL, 1.3 g) was separated on MCI to yield epigallocatechin-(4β→2)-phloroglucinol (subfractions 37–53, 1170 mg).

Gallocatechin-(4α→2)-phloroglucinol was isolated from Fraction 6 (5800–6100 mL, 250 mg) by MCI-gel chromatography (subfractions 42–60, 193 mg).

Fraction 7 (6100–6600 mL, 355 mg) was subjected to chromatography on MCI-gel elution to yield epigallocatechin-(4β→8)-catechin (subfractions 31–39, 67 mg) and epigallocatechin-(4β→8)-epigallocatechin (subfractions 47–52, 112 mg).

Fraction 10 (7830–8200 ml, 45 mg) was separated as above to yield epigallocatechin-(4β→8)-gallocatechin-(4β→2)-phloroglucinol (subfractions 39–45, 19 mg).

The flavan-3-ols and the isolated phloroglucinol adducts were characterized by 1D-, 2D-NMR, circular dichroism spectroscopy (CD), optical rotation and MALDI-TOF-MS of the corresponding derivatives obtained as peracetates in comparison with authentic samples and published values [16–23].

### Epigallocatechin-(4β→8)-epigallocatechin-(4β→2)-phloroglucinol (1):

Fraction 9 (7300–7830 mL, 192 mg) obtained from the Sephadex LH-20 column as described above was subjected to chromatography on MCI-gel CHP 20P (25 × 450 mm) with a 20–60% MeOH linear gradient (17 mL/subfraction) to yield an amorphous powder (subfractions 31–41, 112 mg) **1**: [α]^20^ = +99.3° (c0.10, MeOH). 60 mg were acetylated to give **1a**: MALDI-TOF-MS: [M+Na]^+^ *m*/*z* 1384. ^1^H NMR (CDCl_3_, 600 MHz, δ r = rotameric form): δ 1.21–2.41 (m, Ac), 4.38 (brs, 1H, H-4 F), 4.43 (brs, 1H, Hr-4 F), 4.47 (d, 1H, H-4 C), 4.70 (brs, 1H, Hr-4 C), 4.85 (m, 1H, H-2 F), 4.91 (m, 1H, H-3 C), 5.03 (m, 1H, H-3 F), 5.30 (brs, 1H, Hr-2 C), 5.33 (m, 1H, Hr-3 F), 5.44 (m, 1H, Hr-3 C), 5.49 (brs, 1H, Hr-2 F), 5.68 (brs, 1H, H-2 C), 6.03, 6.26, 6.67, 6.74 (all d, *J* = 2.4 Hz, H-6 A, Hr 6 A, H-8 A, Hr-8 A), 6.65 (s, 1H, H-6 D or H-6r D), 6.85 (s, 1H, H-6r D or H-6 D), 6.83, 6.87, 6.94, 7.04 (all d, *J* = 2.5 Hz, H-4 G, Hr-4 G, H-6 G, Hr-6 G), 6.81, 6.89 (all s, 2H, H-2’/H-6’ E), 6.92, 7.08 (all s, 2H, H-2’/H-6’ B). ^13^C NMR (CDCl_3_, 150 MHz): 32.9 (C-4 C), 34.8 (Cr-4 C), 35.3 (C-4 F), 35.8 (Cr-4 F), 70.2 (Cr-3 C), 70.9 (C-3 C), 71.4 (C-3 F)), 71.4 (Cr-3 F), 73.4 (C-2 C), 74.3 (Cr-2 F), 74.7 (Cr-2 C), 75.4 (C-2 F), 107.4, 107.5, 108.4, 109.3 (C-8 A, Cr-8 A, C-6 A, Cr-6 A), 110.5, 110.8 (C-6 D and Cr-6 D), 111.4 (C-4a D), 111.8 (C-4a A), 114.2, 114.4, 114.6, 114.8 (C-4 G, Cr-4 G, C-6 G and Cr-6 G), 118.9, 119.2, 119.9, 120.2 (C-2’/C-6’ E, C-2’/6’ B, Cr-2’/C-6’ E, Cr-2’/6’ B), 151.8 (C-8a D), 152.1 (C-8a A). After the reaction of compound **1** (20 mg) in 0.1 N ethanolic HCl (1 mL) [27], the mixture was concentrated under a stream of N_2_ to dryness and purified on preparative TLC in system A. The main degradation products were further purified on preparative TLC on cellulose (t-BuOH-CH_3_COOH-H_2_O; 60:20.20) to yield epigallocatechin-(4β→2)-phloroglucinol (13.5 mg) and phloroglucinol (6.1 mg).

### Epigallocatechin-(4β→6)-epigallocatechin-(4β→2)-phloroglucinol (2):

Fraction10 (7830 mL–9300 mL, 107 mg) obtained from the Sephadex LH-20 column as described above was subjected to chromatography on MCI-gel CHP 20P (25 × 450 mm) with a 10–50% MeOH linear gradient (17 mL/subfraction) to yield an amorphous powder (subfractions 56–62, 37 mg) **2**: [α]^20^ = +179° (c 0.17, MeOH). 20 mg were acetylated to give **2a**: MALDI-TOF-MS: [M+Na]^+^ *m*/*z* 1384. ^1^H NMR (CDCl_3_, 600 MHz): δ 1.42–2.43 (m, Ac), 4.25 (brs, 1H, H-4 F), 4.45 (d, 1H, H-4 C), 4.84 (m, 1H, H-3 C), 4.95 (m, 1H, H-3 C), 5.03 (m, 1H, H-3 F), 5.40 (brs, 1H, H-2 C), 5.68 (brs, 1H, H-2 F), 6.60 (d, *J* = 2.2 Hz, 1H, H-6), 6.62 (d, *J* = 2.2 Hz, H-8 A), 6.80 (s, 1H, H-6 D), 6.76 (d, *J* = 2.4 Hz, H-4 G or H-6 G), 6.88 (d, *J* = 2.4 Hz, 1H, H-6 G or H-4 G), 7.16 (s, 2H, H-2’/H-6’ E), 7.29 (s, 2H, H-2’/H-6’ B). ^13^C NMR (CDCl_3_, 150 MHz): 32.7 (C-4 (C), 33.0 (C-4 F), 70.6 (C-3 F), 71.2 (C-3 C), 73.4 (C-2 (F), 73.6 (C-2 C), 107.5 (C-6 A), 107.7 (C-8 A), 109.7 (C-6 D), 111.6 (C-4a D), 111.9 (C-4a A), 114.4 (C-4 G or C-6 G), 114.6 (C-6 G or C-4 G), 119.2 (C-2’/C-6’ E), 119.6 (C-2’/C-6’ B) 151.6 (C-8a D), 152.3 (C-8a A). After the reaction of compound **2** (10 mg) in 0.1 N ethanolic HCl (1 mL) [27], the mixture solution was concentrated under a stream of N_2_ to dryness and purified on preparative TLC in system A. The main degradation products were further purified on preparative TLC on cellulose (t-BuOH-CH_3_COOH-H_2_O; 60:20.20) to yield epigallocatechin-(4β→2)-phloroglucinol (2.4 mg) and phloroglucinol (4.1 mg).

## Figures and Tables

**Tab. 1.** Antioxidant activity and proanthocyanidin (PAC) content of the isolated fractions from *Ginkgo biloba* leaf crude extract.

**Fraction**	**PAC concentration (mg cyanidin /g of fraction)**	**Antioxidant activity (mmol Fremy’s salt reduced/g of fraction)**	**Percentage of antioxidant activity**
Crude dried extract	660.27 ± 7.82	65 ± 1.9	100.0 %
Ethyl-acetate (EAF)	15.52 ± 0.12^a^	33.5 ± 1.1	51.5 %
Methanol-H_2_O (MWF)	73.23 ± 1.77	7.0 ± 0.1	10.8 %
Acetone-H_2_O (AWF)	571.52 ± 6.43	24.5 ± 0.3	37.7 %

a Data are mean ± SD obtained from three different assays.
